# Breast carcinomas associated with microglandular adenosis are linked to germline alterations in homologous recombination-deficiency genes

**DOI:** 10.21203/rs.3.rs-6680831/v1

**Published:** 2025-06-11

**Authors:** Christopher J. Schwartz, Iskender Genco, Matteo Repetto, Daniel Muldoon, Andrea Gazzo, Panieh Terraf, Anne Grabenstetter, Dara Ross, Hong Zhang, Diana Mandelker, Simon Powell, Britta Weigelt, Chaitanya Bandlamudi, Edi Brogi, Fresia Pareja, Hannah Y. Wen

**Affiliations:** Memorial Sloan Kettering Cancer Center; Memorial Sloan Kettering Cancer Center; Memorial Sloan Kettering Cancer Center; Memorial Sloan Kettering Cancer Center; Memorial Sloan Kettering Cancer Center; Memorial Sloan Kettering Cancer Center; Memorial Sloan Kettering Cancer Center; Memorial Sloan Kettering Cancer Center; Memorial Sloan Kettering Cancer Center; Memorial Sloan Kettering Cancer Center; Memorial Sloan Kettering Cancer Center; Memorial Sloan Kettering Cancer Center; Memorial Sloan Kettering Cancer Center; Memorial Sloan Kettering Cancer Center; Memorial Sloan Kettering Cancer Center; Memorial Sloan Kettering Cancer Center

**Keywords:** triple-negative breast cancer, microglandular adenosis, homologous recombination-deficiency, BRCA1, germline, PARPi therapy

## Abstract

Invasive breast carcinomas associated with microglandular adenosis (IBC-MGA) represent a rare and poorly characterized form of triple-negative breast cancer (TNBC). We analyzed clinical, pathological, and germline genetic data from 38 patients, including 34 IBC-MGAs and 4 in situ cases. Germline pathogenic or likely pathogenic variants in homologous recombination-deficiency (HRD) genes were found in 42% (16/38) of patients, predominantly in BRCA1 (81%, 13/16). Most tumors were grade 3 invasive ductal or metaplastic carcinomas with limited tumor-infiltrating lymphocytes. No significant clinicopathologic differences were observed between germline HRD-associated and sporadic cases. Paired tumor-normal targeted sequencing revealed frequent TP53 mutations and high HRD scores. These findings underscore the relationship of breast carcinomas associated with MGA with HRD-related germline variants and highlight the potential for targeted therapeutic strategies and the importance of genetic testing in this rare subset of TNBC.

## INTRODUCTION

Microglandular adenosis (MGA) of the breast is a rare and unusual glandular proliferation notable for its lack of a myoepithelial cell layer and is regarded as a non-obligate precursor of triple-negative breast cancer (TNBC)^1–4^. Breast carcinomas (BCs) arising in MGA span a histologic spectrum that includes *in situ* and invasive carcinoma, commonly referred to as breast carcinoma associated with MGA ^5–7^.

Invasive carcinoma associated with MGA (IBC-MGA) typically exhibits a triple-negative phenotype and most commonly corresponds to the histologic subtypes of invasive carcinoma of no special type (IDC-NST), acinic cell carcinoma, or matrix-producing metaplastic carcinoma^8–14^. We, along with other individual case reports, have observed MGA or IBC-MGA in *BRCA1* germline mutation carriers, prompting us to investigate the potential association between IBC-MGA/MGA and *BRCA1* germline mutations^15–16^ and homologous recombination-deficiency (HRD), a process underlying a subset of breast cancers. HRD, due to mutations in canonical HRD genes, such as *BRCA1, BRCA2*or *PALB2,* impairs the error-free repair of DNA double-strand breaks, resulting in genomic instability and tumorigenesis^17–19^. Defining HRD germline predisposition in IBC-MGA would prove invaluable from the therapeutic perspective, given that patients with HRD BCs exhibit benefit from PARP inhibitor (PARPi) therapy, which induce synthetic lethality by blocking single-strand DNA repair pathways^20^. This study aims to determine the hereditary risk predisposition in patients with IBC-MGA and *in situ* BC associated with MGA (BCis-MGA) through the genetic profiling of these cases.

## RESULTS

### Study cohort

Our cohort included thirty-eight patients diagnosed with either IBC-MGA (n=34) or *in situ* BC-MGA (BCis-MGA; n=4) who had previously undergone germline genetic testing (Table 1 and Supplementary Table S1). The median age at diagnosis across the cohort was 46 years (range: 26–77). The most common clinical presentation was a mammographically detected (34%, 13/38) or palpable mass (34%, 13/38). Magnetic resonance imaging (MRI) revealed mass or non-mass enhancement in nine patients (24%, 9/38). Additionally, three patients (8%) presented with mammographically detected calcifications. A personal history of BC was reported in 24% of patients (9/38), including one patient with synchronous contralateral IDC-NST and one patient with synchronous ipsilateral ductal carcinoma *in situ* (DCIS). Seven patients had a history of multiple malignancies (18%, 7/38). Non-breast malignancies included tubo-ovarian high-grade serous carcinoma (n=3), uterine carcinoma (n=2), melanoma (n=2), lung cancer (n=1), colon cancer (n=1) and meningioma (n=1; Supplementary Table S1). Notably, a family history of BC was reported in 63% (24/38) of patients with 37% (14/38) of them having a first-degree relative with BC (Table 1 and Supplemental Table S1).

On germline genetic testing, 42% (16/38) of patients were found to carry a germline pathogenic or likely pathogenic variant (P/LPV) in an HRD gene. Most of them affected *BRCA1* (81%, 13/16), including recurrent P/LPVs, such as p.Gln1756fs in four patients and p.Ser267Lysfs in two patients. Besides *BRCA1*, other P/LPVs affected *BRCA2* (13%, 2/16) and *PALB2* (6%, 1/16) (Supplementary Table S1). Two patients (5%) carried a germline alteration in HRD genes classified as a variant of unknown significance (VUS), including mutations in *BRCA1* p.Thr1691 (Case 16) and *BARD1* p.Ile764Thr (Case 38).

### Clinicopathologic characteristics

**Figure 1:**
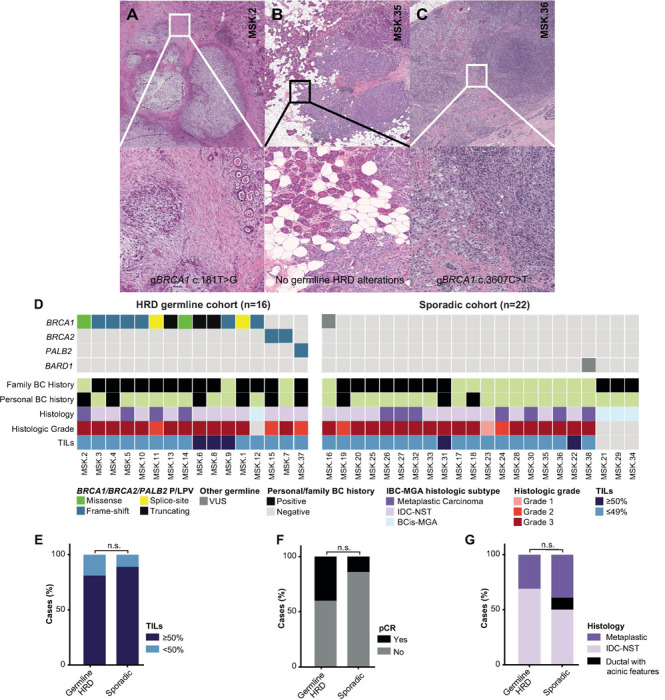
Histologic and clinicopathologic features of breast carcinomas associated with microglandular adenosis Histologic features of invasive breast carcinomas associated with microglandular adenosis (IBC-MGA). (A) Case 2, a 46-year-old *BRCA1* c.181T>G carrier with matrix-producing metaplastic carcinoma associated with atypical microglandular adenosis (B). Case 35, a 32-year-old with invasive carcinoma with acinic cell morphology and associated with atypical microglandular adenosis. Prominent granules in acinic cell-like areas that merge with invasive carcinoma (C) Case 6, a 47-year-old *BRCA1* c.3607C>T carrier with invasive ductal carcinoma of no special type with associated in situ breast carcinoma associated with MGA (BCis-MGA) and atypical microglandular adenosis (2X) Areas of invasion and BCis-MGA merge imperceptibly (D) Oncoplot illustrating the germline pathogenic/likely pathogenic variants in patients with IBC/BC-MGA, along with their associated clinicopathologic characteristics. Stacked bar plots showing comparison of clinicopathologic features between HRD germline carriers and sporadic tumors, including tumor infiltrating lymphocytes (TILs; E), % of complete pathologic response (pCR) to neoadjuvant chemotherapy (F), and histologic type (G). Abbreviations: homologous recombination deficiency, HRD; variant of unknown significance, VUS; invasive breast carcinoma associated with microglandular adenosis; invasive carcinoma of no special type, IDC-NST; in situ breast carcinoma associated with microglandular adenosis, BCis-MGA; tumor infiltrating lymphocytes; TILs, pathologic complete response, pCR.

Histologically, most BC in this study were IBC-MGA (89%, 34/38); four cases (11%; 4/38) corresponded to BCis-MGA (Table 1). The median untreated invasive tumor size was 15 mm (range 2 to 55 mm). The cohort was enriched for matrix-producing metaplastic carcinomas, which constituted almost a third of the cases in the study (32%, 11/34, Fig. 1a). Most, however, were IDC-NST (59%, 20/34), with two tumors showing acinic-cell morphology (6%, 2/34; Fig. 1b). One tumor was a spindle cell metaplastic carcinoma (3%, 1/34). All IBC-MGA were ER-negative and HER2-negative (Supplementary Table S1), and the majority showed Nottingham histologic grade 3 (82%, 28/34; Fig. 1c). Lymphovascular invasion was present in 18% (7/38) of cases, and 21% had lymph node metastases (7/33). The majority of tumors (85%, 29/34) demonstrated less than 50% stromal tumor-infiltrating lymphocytes most of which exhibited fewer than 20% stromal TILs (82%, 28/34).

Most patients underwent mastectomy (55%, 21/38), while 45% (16/38) had breast conserving surgery. Eleven patients with IBC-MGA (32%, 11/34) received neoadjuvant chemotherapy (NACT), and pathologic complete response (pCR) was observed in 18% (2/11) of them (Table 1).

Most IBC-MGA received adjuvant treatment, including radiotherapy (68%, 21/31) and chemotherapy (77%, 24/31). Six patients with IBC-MGA experienced disease recurrence, including one local recurrence (3%, 1/31) and five distant metastases (16%, 5/31).

One of four patients with BCis-MGA received radiotherapy and none received chemotherapy. No patients with BCis-MGA (n=4) experienced disease recurrence (Supplementary Table S1).

### Clinicopathologic features of BC-MGA in patients with germline HRD alterations and sporadic cases

Given the distinct clinicopathologic features associated with HRD in BCs and the high prevalence of germline HRD variants in BCs associated with MGA, we conducted a secondary analysis to compare cases with germline HRD alterations (gHRD, n=16) to sporadic cases (n=22; Figs. 1d; Supplementary Table S2). Patients with gHRD and had a comparable median age at presentation to sporadic cases (45 years (range: 26–67) vs. 46.5 years (range 32–77) (*P*-value = 0.9).

Although two patients with IBC-MGA in the gHRD group achieved pCR compared to none in the sporadic group, it this finding did not achieve statistical significance (40%, 2/5 vs. 0%, 0/6; *P*-value = 0.4). No other clinicopathologic factors were found to be distinct between the two cohorts (Figs. 1e-1g; Supplementary Table S2), although interpretation is limited due to the small cohort size.

### Somatic genetic alterations and HRD features of IBC-MGA

Panel sequencing targeting up to 505 genes (MSK-IMPACT) was performed on six IBC-MGA cases, including four primary tumors (two matrix-producing metaplastic carcinomas, one spindle cell metaplastic carcinoma, and one IDC-NST and two metastatic tumors (one matrix-producing metaplastic carcinoma and one IDC-NST). Among these, 2 of 6 cases were associated with P/LPV germline alterations: Case 5, a metastatic matrix-producing metaplastic carcinoma, was a *BRCA1* germline mutation carrier, and Case 37, a metastatic IDC-NST, was from a *PALB2* germline mutation carrier. All other tumors subjected to panel sequencing lacked P/LP germline alterations in HRD genes, including Case 38, a primary metaplastic spindle cell carcinoma.

**Figure 2:**
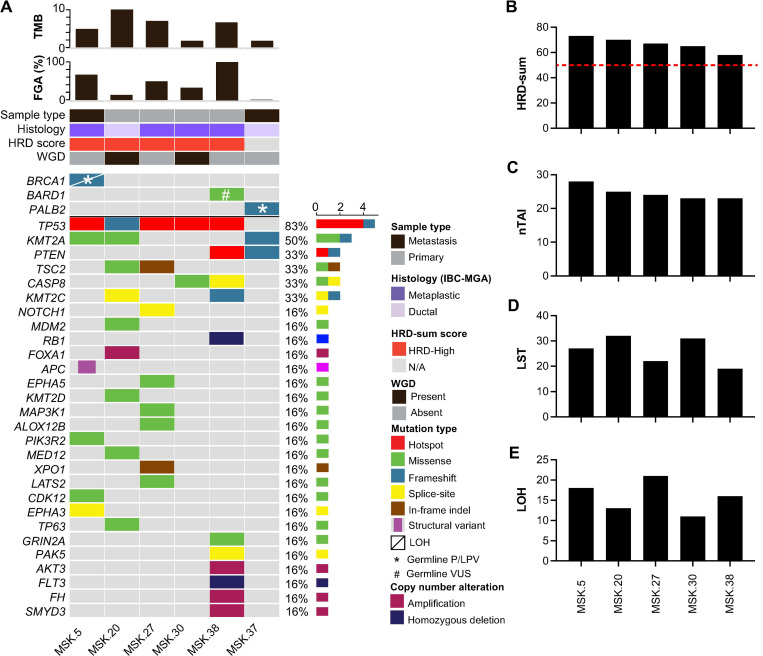
Somatic genetic alterations in invasive breast carcinomas associated with microglandular adenosis. Heatmap depicting individual cases are displayed in columns, while genes are organized in rows. Phenobars in the right panel indicate the invasive carcinoma subtype, the sample type sequenced, identified genetic alterations and IMPACT-HRD scores (A). IMPACT-HRD scores across cases (B), including number of telomeric imbalances, nTAI (C), large scale state transitions, LST(D) and genome wide loss of heterozygosity, LOH (E). Abbreviations: tumor mutational burden, TMB; fraction of genome altered, FGA; homologous recombination deficiency, HRD; whole genome duplication, WGD; invasive breast carcinoma associated with microglandular adenosis, IBC-MGA; loss of heterozygosity, LOH; pathogenic/likely pathogenic variant, P/LPV, variant of unknown significance, VUS.

The median tumor mutational burden and fraction of genome altered were 5.8 mut/Mb (range, 1.8–10) and 41% (range, 1–99), respectively. Whole-genome duplication was observed in 2/6 (33%) cases (Fig. 2a). Five of six BCs (83%, 5/6) harbored pathogenic *TP53* mutations. Recurrent alterations were also observed in genes involved in chromatin remodeling (67%, 4/6), particularly *KMT2A* (50%, 3/6) and *KMT2C* (33%, 2/6) (Fig. 2a). Two BCs (33%, 2/6) harbored alterations in either *PTEN* and/or *CASP8.* No somatic alterations affecting HRD genes were observed.

Given the observed prevalence of germline HRD alterations in IBC-MGAs, we next investigated whether these tumors exhibited genomic features associated with HRD defects. We utilized an IMPACT-HRD score^21^, which incorporates genome wide LOH, nTAI, and LST, with a threshold of ≥50 considered indicative of the HRD phenotype (see Methods). In the five IBC-MGA cases analyzed (100%, 5/5; Fig. 2b-2e) all exhibited high HRD scores (≥50, see Methods), including Case 5, which harbored a germline *BRCA1* mutation and somatic LOH.

Although this analysis was conducted on a limited number of cases, these findings align with the high prevalence of germline HRD alterations in this cohort and further support a key role for HRD in the pathogenesis of IBC-MGA.

## DISCUSSION

Through germline profiling, this study identified a novel association between IBC-MGA and germline alterations in HRD-associated genes, found in 42% of cases. In line with these findings, all IBC-MGA cases with available paired tumor/normal targeted sequencing data exhibited HRD genomic features and were classified as HRD. This association has significant therapeutic implications, as a subset of patients with IBC-MGA may benefit from PARPi. These findings highlight the importance of germline genetic testing in IBC-MGA patients to guide treatment decisions.

Although all IBC-MGA cases exhibited HRD genomic features, only one case harbored a pathogenic germline *BRCA1* P/LPV with biallelic LOH. No somatic alterations in other core HRD genes were identified in the remaining cases. The underlying genomic basis for HRD was not elucidated in these cases. It is plausible that IBC-MGA may harbor genetic or epigenetic alterations in HRD pathway genes that are not detectable by targeted sequencing, such as complex structural rearrangements or epigenetic alterations.

Despite a range of histologic phenotypes including a notable enrichment in metaplastic carcinomas, IBC-MGA appears to have lower overall levels of tumor-infiltrating lymphocytes (TILs), with only 5% of cases showing >50% TILs^22^, compared to triple-negative IDC-NST. Additionally, the pathologic complete response (pCR) rate was slightly lower at 18%, compared to 28% observed in conventional TNBC^23^. Both examples of pCR occurred in patients with P/LPV variants in HRD genes. Further studies with larger sample sizes are recommended to verify this observation. Interestingly, all four patients with BCis-MGA remained disease-free at the time of the study. This supports the notion that in the absence of frankly invasive carcinoma with stromal desmoplasia and inflammation, BCis-MGA may behave akin to ER-negative ductal carcinoma in situ, as previously reported^24^. Nonetheless, the disease recurrence observed in a subset of patients with IBC-MGA (16%) underscores the importance of potential targeted therapy. Given the high rate of germline alterations in HRD genes and HRD genomic features identified in these breast cancers, PARPi may offer potential benefit.

Paired tumor-normal targeted sequencing in six cases, including a gHRD case with a *BRCA1* inactivating mutation, revealed highly recurrent *TP53* somatic mutations in IBC-MGA, akin to both MGA and those reported in conventional TNBC and mammary acinic cell carcinoma (AciCC)^12–13^. Mammary AciCC is a BC that shares significant morphologic and genomic overlap with IBC-MGA, and indeed 6% of IBC-MGA in our study showed acinic cell features. Recent studies support the notion that mammary AciCC is a distinct entity from its salivary gland counterpart, as it lacks the characteristic t(4;9) (q13;q31) genomic rearrangement and NR4A3 overexpression, both of which are hallmark features of salivary gland AciCC^25–26^. Interestingly, P/LPV *BRCA1* germline variants have been identified in patients with AciCC. These observations support the notion that mammary AciCC and IBC/BC-MGA are very closely related lesion or even represent the same entity.

This study has limitations, such as the small cohort size, due to the rarity of IBC-MGA, in which < 100 of these tumors are described in the literature. Additionally, MSK-IMPACT was conducted in only six cases and the MSK-IMPACT panel includes only 505 cancer genes, which targets a predefined set of genes, restricting the identification of a broader group of genetic alterations.

In summary, we identified an association between BC-MGA and germline pathogenic/likely pathogenic variants (P/LPVs) in HRD-associated genes, particularly in *BRCA1.* While IBC-MGA in germline and sporadic contexts shares similar clinical features, these findings may have important implications for future treatment strategies and personalized medicine approaches.

## METHODS

### Study Cohort and Data Collection

The cohort consisted of 38 patients, including 34 patients with IBC-MGA and 4 patients with BCis-MGA over a 24-year period. This study was approved by the Institutional Review Board at Memorial Sloan Kettering Cancer Center (MSK). Clinicopathologic data for all cases were collected, including genetic testing data, patient demographics (age and family history), tumor size, treatment regimens, and outcomes. Individuals with likely pathogenic or pathogenic mutations in cancer susceptibility genes (CAGs) were curated by a board-certified medical geneticist (PT) following the criteria established by the American College of Medical Genetics and Genomics^27^.

### Histopathologic Assessment

Histopathologic assessment of each tumor was evaluated by at least two study breast pathologists (CS and HYW). The assessment of histologic subtype was conducted following the World Health Organization classification criteria^1^. Estrogen receptor (ER) and human epidermal growth factor 2 (HER2) were obtained from pathology reports and previously assessed according to the American Society of Clinical Oncology/College of American Pathologists recommendations^28^. The extent of tumor-infiltrating lymphocytes (TILs) was determined in all invasive tumors following the criteria set forth by the International TILs Working Group^22^.

### Statistical Analysis

Statistical analyses were conducted using R version 1.2. Fisher’s exact test was used for categorical variables, and continuous variables were compared using the student’s t-test. All tests were two-sided, with a P-value of less than 0.05 considered statistically significant.

### Next-Generation Sequencing and HRD score analysis

We investigated somatic genetic alterations in subset of cases (n=6) that had been previously subjected to the FDA-approved paired tumor-normal targeted sequencing using the MSK Integrated Mutation Profiling of Actionable Targets (MSK-IMPACT) assay in the clinical setting^29^. Targeted sequencing data of these cases was retrieved from cBioPortal^30^ and retrospectively analyzed. Loss of heterozygosity of the wild type allele (LOH) of affected germline HRD genes was assessed using FACETS^31^. We inferred previously established measures of HRD phenotype using an algorithm optimized for MSK-IMPACT^32^. In brief, the IMPACT-HRD score is computed as an unweighted sum of the genome-wide loss of heterozygosity (LOH), the number of telomeric imbalances (nTAI) and large-scale transitions (LST). The IMPACT-HRD scores were strongly correlated and concordant with Myriad’s Genomic Instability Scores in a cohort of germline *BRCA1/2* wild-type ovarian cancer patients who received Myriad testing as well as MSK-IMPACT profiling as part of routine clinical care (data not shown) ^21^. Here, we used a conservative IMPACT-HRD score threshold of 50 to determine a tumor to be HRD-positive. Of note, this is significantly greater than Myriad’s FDA-approved threshold of 42 for ovarian cancer patients and an exploratory threshold of 33 for breast cancer patients^32–33^.

## Supplementary Material

Supplement 1

## Data Availability

Genetic alterations identified by MSK-IMPACT will be available on cBioPortal upon publication.
