# Supplementary material for: Breast carcinomas associated with microglandular adenosis are linked to germline alterations in homologous recombination-deficiency genes
Source: Res Sq. 2025 Jun 11:rs.3.rs-6680831. Preprint. [Version 1] doi: 10.21203/rs.3.rs-6680831/v1 (PMC12204481; doi:10.21203/rs.3.rs-6680831/v1)
Supplement: Supplement 1 [file NIHPPRS6680831V1-supplement-1.pdf]

Tables

Table 1 is available in the Supplementary Files section.

Supplementary Files

This is a list of supplementary files associated with this preprint. Click to download.

- Table1.docx
- SupplementaryTablesS1S2.xlsx
